# The Segment, Slice, and 3D Print (SS3DP) Workflow of 3D Printing Eye Anatomy for Clinicians: A Proof-of-Concept Study

**DOI:** 10.7759/cureus.49614

**Published:** 2023-11-28

**Authors:** Periklis Giannakis, Mohsan Malik

**Affiliations:** 1 Barts and the London School of Medicine and Dentistry, Queen Mary University of London, London, GBR; 2 Adnexal Service, Moorfields Eye Hospital National Health Service (NHS) Foundation Trust, London, GBR

**Keywords:** eye anatomy, image segmentation, slicing, ophthalmology, ‎3d printing

## Abstract

3D printing is becoming increasingly important as time passes, with the latest technologies driving innovation in many fields, including ophthalmology. However, more is needed to know how clinicians can become innovators in their daily practice without needing expert engineering knowledge of the underlying technologies. We aimed to address that shortcoming by developing a pipeline clinicians can use to 3D print. This workflow was named SS3DP: Segment, Slice, and 3D Print. It was tested by fabricating a 3D-printed eyeball. In terms of the results of this work, we observed that the segmentation process was imperfect due to the difficulty of segmenting small structures. The learning curve was steep initially, but the technique improved the more time spent on the segmentation platform. No quantitative analysis was carried out. Innovation in medicine is stifled if its leading participants, clinicians, cannot engage with it due to a lack of knowledge.

## Introduction

Technological innovation has enabled 3D printing, which has risen in use in the last few years owing to lower entry costs and interest in designing patient-specific treatments at the point of care. In ophthalmology, they have been used to produce ocular implants and prostheses, moulds to guide surgical planning, adapters used to diagnose diseases of the retina and eye models to teach anatomy [1].

3D printed anatomical models have proven utility for medical undergraduate and postgraduate education in replacement of traditional cadaveric dissection. Previous work has focused on 3D printing models of the circle of Willis, the cerebral venous system, and the whole eye, while others have constructed eyes made of glass that were used to teach an ophthalmoscopy [2,3]. Another team constructed eyes from CT images and edited them to highlight critical anatomical structures, which, when 3D printed, proved to be a very accurate representation of the bony and soft anatomy of the orbit but lacked the precision needed to capture fine osteological features of the orbital bones [4]. Nevertheless, despite the lack of limitations, 3D-printed eyes from cadaveric prosections were formally assessed to be of sufficient quality for postgraduate training by the Royal Australian and New Zealand College of Ophthalmologists [5]. Further application includes 3D printing surgical simulation models, such as strabismus to lessen the learning curve [6].

Although early adoption and application of 3D printing in ophthalmology are very exciting, but are not easily accessible due to the specialist skills required and lack of knowledge of fabrication methodologies. Therefore creating a reliance on industry or resourceful academic institutions to lead. This creates an elite model thereby limiting wider benefit. We report a 3D printing pipeline to improve access to anatomical modelling and printing using real-life data from imaging modalities such as CT or MRI.

## Technical report

Method

Development of Eye Model (SS3DP) Method

**Figure 1:**
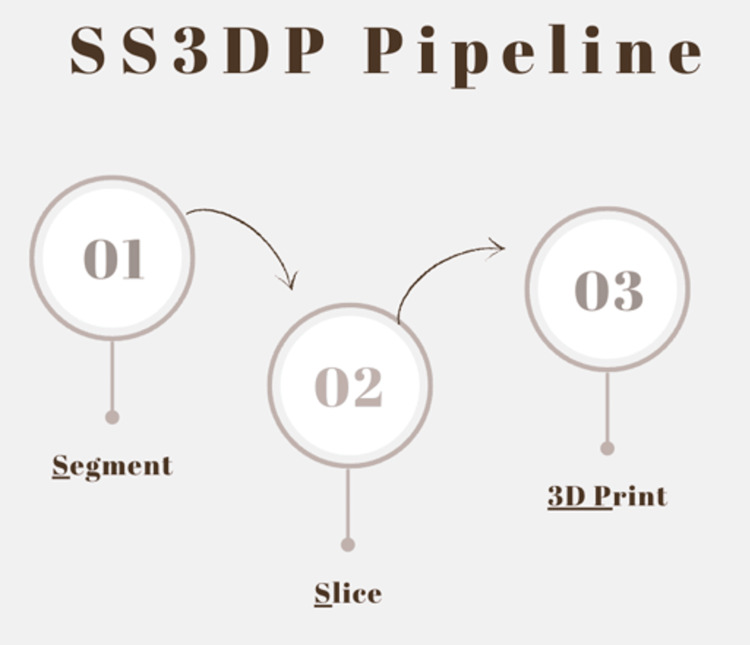
This flowchart depicts the pipeline and workflow. Open source software (3D slicer version: 5.2.2) is used to segment, and UltiMaker Cura (UltiMaker B.V., NY, USA) to slice images, preparing the output for 3D printing.

We developed the Segment, Slice and 3D Print (SS3DP) pipeline using open-source software (Figure 1) [7-9]. The MRI images used in this example were freely available on the Slicer website (https://www.slicer.org/) [7].

Segmentation

Digital Imaging and Communications in Medicine (DICOM) is an international technical standard for the digital storage and transmission of medical images, which are composed of stacked 2D images. 3D printing from DICOM images needs to be segmented into a format compatible with the 3D printer. DICOM images are now commonly segmented into a 3D computer-aided design (CAD) format, serving as intermediate data for further processing, including setting the region of interest (ROI). Among the various file formats for 3D CAD data, the Standard Tessellation Language (STL) file format is the most widely used for 3D printing.

**Figure 2:**
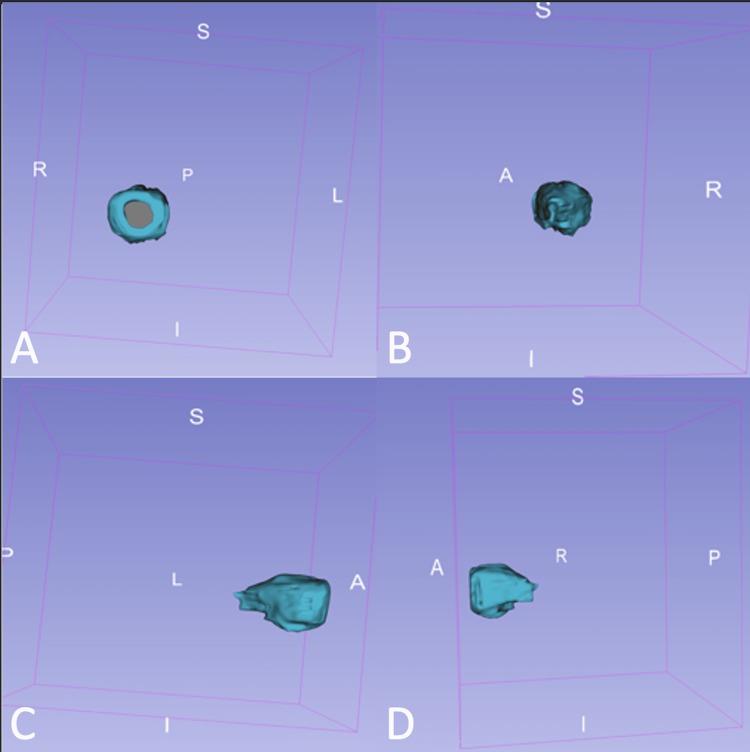
This figure shows the segmented eyeball with the overlying connective tissue. These images were taken after the segmentation process was completed on the 3D Slicer.

We used an open source software for the segmentation process utilising two pre-installed modules, namely the volume rendering and segmentation editor. Initially, we volume-rendered the three images to allow us to visualise the MRI images. We then adjusted the display shift to higher values to make the human head mould more recognisable. The segmentation editor was then used to create two segmentations, eye and connective tissue. We used the level tracing function to automatically capture most of the areas of interest. The draw and erase function allows us to tailor the segmentation to define precision and eliminate artefacts. Once the segmentation of each structure was complete, we exported it into STL format and merged the segmented structures into one (Figure 2, Video 1).

**Video 1:** Segmentation tutorial

Slice

Slicing involves transforming the 3D model file into a machine-readable language, known as the G-Code file. The printer can only execute successful prints after interpreting this specific machine language.

**Figure 3:**
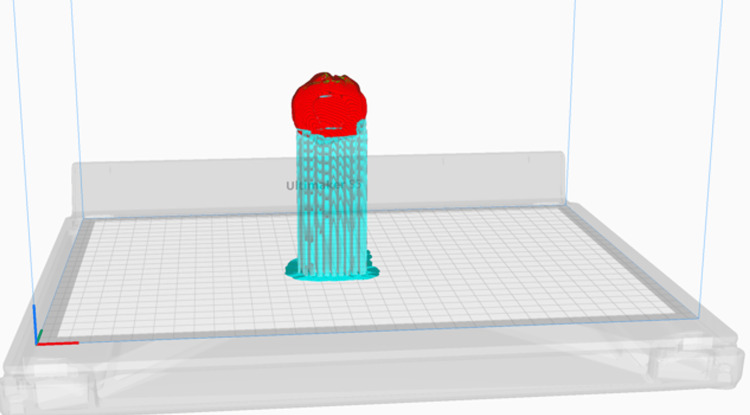
This figure shows the sliced eye model Red represents the eye model. Blue represents the support material required to print the eye model whilst maintaining structure.

We imported the STL file on UltiMaker Cura (UltiMaker B.V., NY, USA), which enabled us to reconstruct the print instructions. We used generic tough polylactic acid material with print core AA 0.4 and generic polyvinyl alcohol AA 0.4 for the second extruder, with 20% infill to provide support. Alternative print core, such as AA 0.8, may offer speed for printing. However, we selected 0.4 which offered the best resolution of 0.1 mm required. We set the profile to Visual to maximise the accuracy of the 3D-printed eye model. We then sliced it and exported the sliced eye model into the UltiMaker Format package (.ufp) (Figure 3).

3D Print

**Figure 4:**
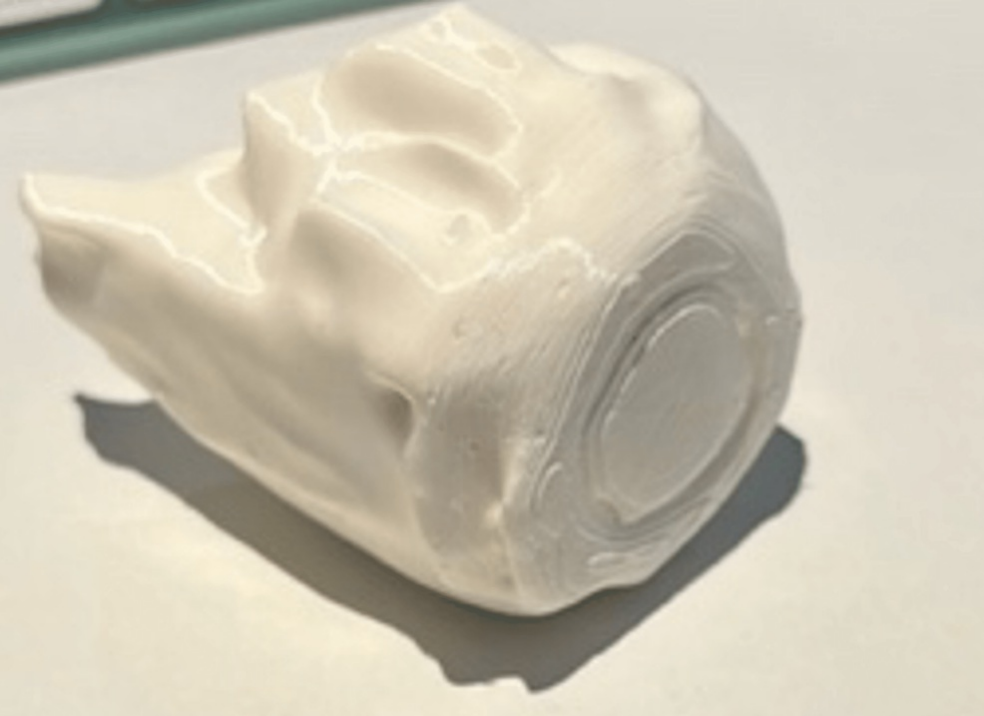
This figure shows the 3D-printed eye model.

UFP files can be then imported and executed by the 3D printer (we used UltiMaker S5). After production, we let the model dry and removed the support material (Figure 4). On physical inspection of the 3D printed model, areas of smoothness were observed with some rough surfaces owing to imperfect segmentation. Focal heating to the areas can utilised to manually sculpt the model and reform the material. Alternatively, further refinement of the segmentation can be performed if multiple printing is required.

## Discussion

In this report, we developed an SS3DP workflow that clinicians can use to 3D print eye models originating from 3D imaging techniques, such as MRI or CT, which cost under £5 to produce.

The limitation of the resolution of the model is dependent on the primary source quality and segmentation process. Higher-quality images or using a combination of 3D imaging modalities can increase the accuracy of the 3D models. This may be accomplished using the logical operators function on the 3D slicer, which can combine segmentations. We used generic MRI scans for the proof of concept that was freely available, rather than dedicated orbital or ocular imaging, which would have provided better resolution.

Slicing and 3D printing depend highly on segmentation, so the better the segmentation, the better its by-products. Our model had imperfections, partly due to the manual and imprecise segmentation. We could overcome the challenges by physically sculpting or refining the 3D model with manual slice editing; the latter, which improves print quality, is more time-consuming. The learning curve for segmentation is steep, for this reason, we present a video tutorial. With the advancement of segmentation, clinicians who strive for very high anatomical accuracy could consider using automatic segmentation like the one used by Ciller et al. that segmented the sclera, cornea, vitreous body and lens [10]. Alternatively, volumetric heatmaps can quantify segmentation, showing mismatches to refine the model. Although no formal metrics exist, they would yield higher accuracy if used. However, these are helpful markers that can be studied and used with caution to help delineate clinically significant differences [11]. Looking ahead, multiple segmentations may also help get the best outcomes. The simultaneous Truth and Performance Level Estimation (STAPLE) algorithm can combine all different segments of the same structure, which are scored, producing a probabilistic estimation of the actual segmentation [12]. This would be instrumental, especially for applying our pipeline in surgical planning, where accuracy is paramount. The ideal situation would be to have auto-segmented 3D file formats directly from the primary source rather than conversion software.

Using a high-quality 3D printer helps make the process much more accessible but comes with a considerable cost. Although entry costs to 3D printers have fallen drastically over the years, professional 3D printers are still expensive and may not be affordable and cost-effective for every clinician [13]. Professional 3D printing services exist and are more cost-effective if clinicians do not plan to use the technologies extensively.

Several limitations should be considered when interpreting the study results. Firstly, the 3D-printed eyeball wasn't assessed against educational standards for its anatomic accuracy and usefulness. Future studies on this should aim to seek to validate 3D-printed objects against official academic guidelines reporting anatomic accuracy. Secondly, this is a proof-of-concept guide to provide access to 3D model printing using non-dedicated, general, free-to-use DICOM images. We did not quantitatively assess the findings using validated methods which would have otherwise increased the accuracy and precision of the segmentations. Thirdly, this study produced only one eye prototype, but we should have printed the same eye many times to observe if there were any significant visual discrepancies caused by 3D printing.

The pipeline has many implications for nearly all specialities as they can be used to construct anatomical structures for teaching, surgical planning or ocular implants, paving the way to more patient-specific treatments at the point of care [1].

Future work should focus on validating our pipeline and using experts in the field to segment and validate the 3D-printed prototypes. It is also essential for different 3D printers to be used to assess how more affordable options can produce equivalent results because not every clinician can afford to buy a 3D printer that costs £5,000 [13].

## Conclusions

In summary, we developed a pipeline called SS3DP that clinicians can use to 3D print anatomical structures. Although limited to eyes, this study can be used for other systems and other specialities as the principles of the pipeline are universal. This work can be applied to other aspects of ophthalmology, making 3D printing a powerful tool to educate and pre-operative planning.
